# Comparing Telemedicine and In-Person Psychological Interventions for Anxiety: A Systematic Review

**DOI:** 10.7759/cureus.89594

**Published:** 2025-08-08

**Authors:** Mohaned Eltaj Ibrahim, Hanady ME M Osman, Ahmed Mohamed Elamin Mubarak Osman, Nawaf Munis Mofareh Alanazi, Basil Tarig Mohamed Ali, Ikhlas Ibraheem Adam Abdallah, Musab Ibrahim Hussein Mohamed

**Affiliations:** 1 Psychiatry, Public Health Authority, Riyadh, SAU; 2 Quality and Patient Safety, Najran Armed Forces Hospital, Ministry of Defense Health Services, Najran, SAU; 3 General Medicine, Jouf University Medical Services Center, Sakaka, SAU; 4 Nursing, Najran Armed Forces Hospital, Ministry of Defense Health Services, Najran, SAU; 5 General Medicine, Gulf Medical University, Ajman, ARE; 6 Psychiatry, Erada Hospital for Mental Health, Riyadh, SAU; 7 Psychiatry, Ibri Regional Hospital, Ibri, OMN

**Keywords:** anxiety disorders, cognitive behavioral therapy, mental health, systematic review, telehealth, telemedicine

## Abstract

Barriers such as stigma and limited access to care continue to impede treatment for anxiety disorders. Telemedicine has emerged as a promising alternative to in-person psychological interventions, particularly after the COVID-19 pandemic. This systematic review compares the efficacy of telemedicine and in-person therapies for anxiety disorders, evaluating outcomes, patient engagement, and methodological rigor. Following PRISMA 2020 guidelines, we searched PubMed, Scopus, Web of Science, and ClinicalTrials.gov, with the final search conducted in July 2025. Ten studies comparing telemedicine with in-person interventions were included. Risk of bias was assessed using the Cochrane RoB 2 tool for randomized controlled trials and the Newcastle-Ottawa Scale for non-randomized studies. A narrative synthesis was conducted due to heterogeneity. Telemedicine demonstrated non-inferior efficacy to in-person therapy across diverse modalities and outperformed self-help programs. Patient satisfaction and adherence were high, with telehealth groups showing longer retention. Small effect size differences favored in-person therapy for generalized anxiety disorder, but most studies reported comparable outcomes. Risk of bias was low for nine out of ten studies. Telemedicine is a viable alternative to in-person therapy for anxiety disorders, with advantages in accessibility and therapist-guided formats. Future research should address long-term outcomes and equity in delivery.

## Introduction and background

Anxiety disorders are among the most prevalent mental health conditions globally, affecting over 301 million people and leading to substantial personal, societal, and economic consequences [1]. These conditions can significantly impair quality of life, social functioning, and productivity. Psychological interventions, particularly cognitive behavioural therapy (CBT) and other evidence-based approaches, have long been considered first-line treatments for anxiety disorders [2]. However, access to such treatments is often hindered by factors like therapist shortages, stigma, geographical limitations, and scheduling conflicts [3].

Telemedicine has emerged as a viable solution to overcome these barriers. Enabled by digital advancements and expanded telecommunication infrastructure, psychological therapies can now be delivered remotely through videoconferencing, internet-based platforms, and other telehealth technologies [4]. The COVID-19 pandemic further accelerated the adoption of telehealth globally, emphasizing its potential to deliver mental health care effectively while improving access and continuity of services [5].

While telemedicine has been applied across a spectrum of mental health conditions - including anxiety, depression, and obsessive-compulsive disorder (OCD) - this review focuses specifically on anxiety disorders to maintain clarity and clinical relevance. Previous reviews have often aggregated outcomes across broad psychiatric populations or emphasized general telehealth utility, limiting the applicability of findings to anxiety-focused care [4,6].

Given the rapid growth in telemedicine use and the continued relevance of in-person care, a direct comparison of these two modalities is crucial. Therefore, this systematic review aims to synthesise existing evidence comparing the effectiveness of telemedicine and in-person psychological interventions in reducing anxiety symptoms. By evaluating outcomes, intervention types, and potential moderating factors, this review seeks to provide an evidence-based understanding of their comparative value, ultimately guiding clinicians, researchers, and policymakers in optimizing mental health care for individuals with anxiety disorders.

## Review

Methods

Review Protocol

This systematic review was conducted in accordance with the Preferred Reporting Items for Systematic Reviews and Meta-Analyses (PRISMA) 2020 guidelines to ensure methodological rigour, transparency, and reproducibility [7].

Eligibility Criteria

This systematic review included studies that directly compared telemedicine-based psychological interventions with in-person psychological interventions for the treatment of anxiety disorders. Eligible studies were randomized controlled trials (RCTs), non-randomized controlled studies, and cohort studies involving human participants diagnosed with any anxiety disorder based on standard diagnostic criteria such as DSM or ICD classifications. Studies were included if they reported quantitative anxiety outcomes measured using validated scales. There were no restrictions on participant age, sex, or geographical location. Studies not published in English, review articles, conference abstracts, commentaries, and protocols were excluded.

Information Sources

A comprehensive literature search was conducted using the following electronic databases: PubMed, Scopus, Web of Science, and ClinicalTrials.gov. These databases were selected to ensure broad coverage of published and registered trials relevant to psychological interventions in anxiety. The final search was performed in July 2025.

Search Strategy

Search strategies were developed using relevant MeSH terms and free-text keywords related to “telemedicine,” “telepsychology,” “teletherapy,” “in-person therapy,” “face-to-face therapy,” and “anxiety disorders.” Boolean operators were applied to combine concepts appropriately. The full search strategies for each database are available in the Supplementary Material (see Appendices) to facilitate reproducibility and transparency.

Selection Process

All identified records were imported into EndNote for duplicate removal. Two independent reviewers screened titles and abstracts for eligibility, followed by full-text screening of potentially relevant studies. Discrepancies were resolved through discussion or adjudication by a third reviewer. The study selection process is illustrated in the PRISMA flow diagram.

Data Collection Process

A standardized data extraction form was developed in Excel. Two reviewers independently extracted data on study characteristics, participants, interventions, comparators, outcome measures, results, and risk of bias assessments. Any disagreements in data extraction were resolved through discussion or by consulting a third reviewer to ensure accuracy and consistency.

Data Items

Key data items extracted included first author, publication year, country, study design, sample size, participant characteristics (age, sex, diagnosis), intervention type (telemedicine modality), comparator type (in-person therapy details), duration and frequency of interventions, outcome measures (including scales used for anxiety), and main findings including effect sizes or p-values where available.

Study Risk of Bias Assessment

The Cochrane Risk of Bias tool version 2 (RoB 2) [8] was used to assess the methodological quality of included RCTs, evaluating domains such as randomization process, deviations from intended interventions, missing outcome data, measurement of outcomes, and selection of reported results. For non-randomized and observational studies, the Newcastle-Ottawa Scale (NOS) [9] was employed to evaluate the risk of bias across selection, comparability, and outcome assessment domains. Assessments were conducted independently by two reviewers, with disagreements resolved through discussion.

Effect Measures

For each included study, the primary effect measure extracted was the mean difference or standardized mean difference in anxiety outcomes between telemedicine and in-person intervention groups, along with reported p-values or confidence intervals.

Synthesis Methods

Given the expected heterogeneity in intervention modalities, outcome measures, study designs, and participant characteristics, as well as anticipated differences in delivery platforms, therapist qualifications, and session structures, meta-analysis was not conducted. A narrative synthesis approach was employed to summarize findings systematically, highlighting similarities and differences across studies and exploring potential sources of heterogeneity to inform clinical implications. This decision was made to avoid inappropriate pooling of highly diverse studies, which could lead to misleading or spurious conclusions.

Reporting Bias Assessment

Due to the narrative nature of synthesis and absence of meta-analysis, formal statistical assessment of publication bias, such as funnel plot asymmetry, was not applicable. However, potential for publication bias was discussed based on the pattern of results across studies.

Results

Study Selection Process

The study selection process followed the PRISMA guidelines and is summarized in the attached flowchart. Initially, 185 records were identified through systematic searches across multiple databases, including PubMed (n=64), Scopus (n=59), Web of Science (n=41), and ClinicalTrials.gov (n=27), supplemented by 14 additional records from citation searching. After removing 92 duplicate records, 93 studies underwent title and abstract screening, of which 63 were excluded for irrelevance.

**Figure 1:**
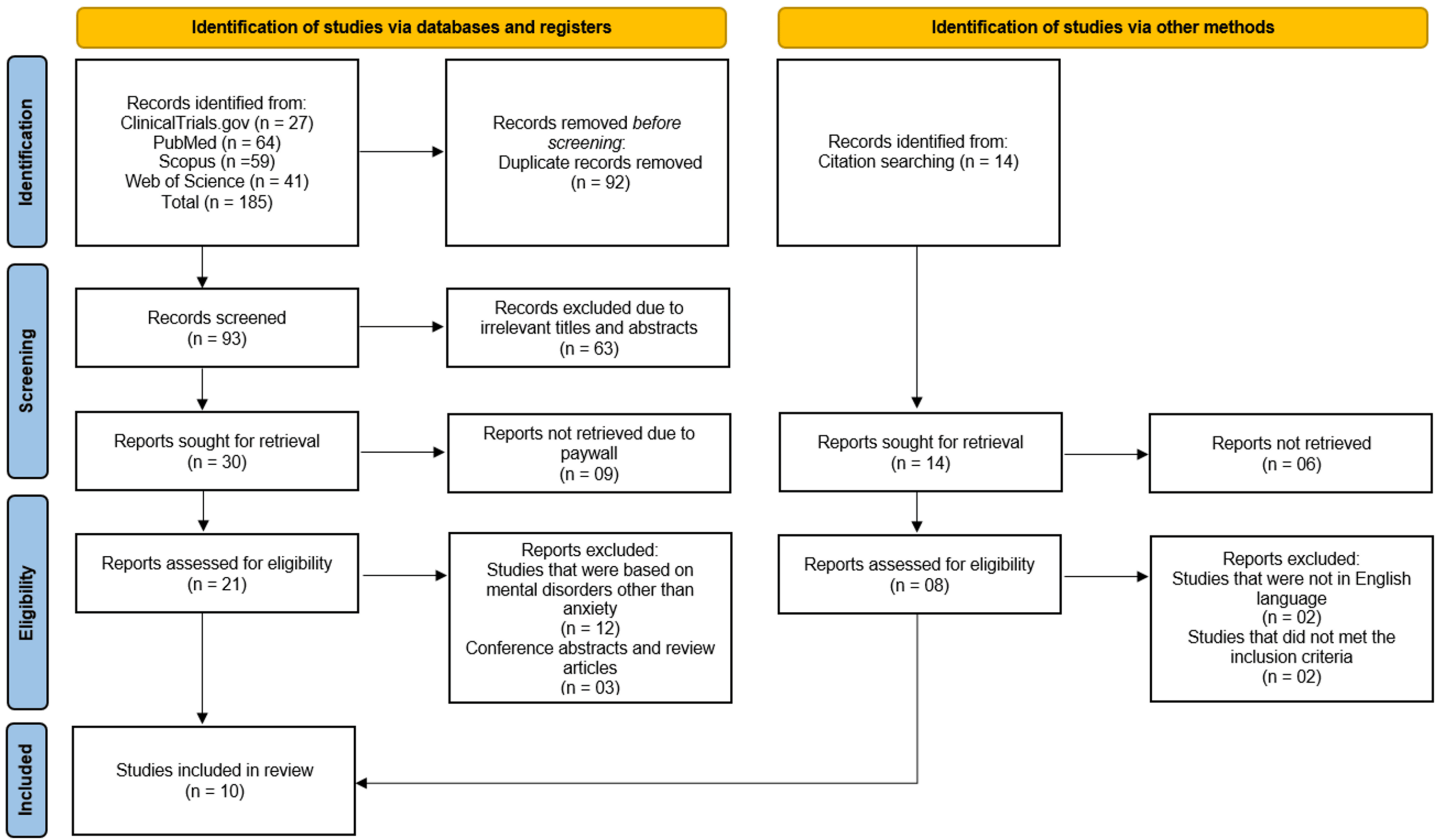
PRISMA Flow Diagram of Study Selection Process

A total of 30 full-text reports were sought for retrieval, but nine were inaccessible due to paywalls, leaving 21 reports for eligibility assessment. Of these, 12 were excluded for focusing on mental disorders other than anxiety, three were conference abstracts or review articles, and two were non-English publications. Additionally, two studies did not meet the inclusion criteria. Ultimately, 10 studies [10-19] were included in the systematic review for qualitative synthesis (Figure 1).

Overview of Included Studies

This review included 10 studies [10-19] comparing telemedicine and in-person psychological interventions for anxiety and related disorders (Table 1). The studies spanned diverse geographical locations, including Iran, Japan, Canada, the United States, Oman, the United Kingdom, and Australia, and encompassed various telemedicine modalities such as internet-based CBT, videoconferencing, telephone-delivered CBT, and two-way video treatments. Sample sizes ranged from 22 to 2,384 participants, with study designs including RCTs, non-randomized comparisons, and observational studies. The interventions targeted a broad spectrum of anxiety-related conditions, including social anxiety disorder, generalized anxiety disorder (GAD), panic disorder, OCD, and depression with comorbid anxiety.

**Table 1:** Key Characteristics of Included Studies

Author(s), Year	Country / Setting	Study Design	Sample Size (n)	Participant Characteristics	Intervention Type	Comparator	Duration / Frequency	Outcome Measures	Key Findings / Results
Rad et al. [10], (2024)	Iran / Lorestan province	Pretest-posttest follow-up experimental design	54 adolescents	Adolescents with social anxiety disorder, selected via cluster sampling	Internet-based CBT	Face-to-face CBT and wait-list control	10 weekly sessions, 3-month follow-up	Social phobia, fear of negative evaluation, social interaction anxiety, emotion regulation, physical symptoms, insomnia, social dysfunction, depression symptoms	Both internet-based and face-to-face CBT effectively reduced primary symptoms and secondary symptoms (physical symptoms, insomnia, social dysfunction, depression). Effects were stable at 3-month follow-up.
Kishimoto et al. [11] (2024)	Japan	Pragmatic Randomized Controlled Trial	199	Adults with depressive disorder, anxiety disorder, or obsessive-compulsive disorder in subacute/maintenance phase	Two-way video treatment via smartphones or other devices (≥50% video sessions)	Face-to-face treatment (100% in-person sessions)	24 weeks	Primary: SF-36 Mental Component Summary (MCS) score; Secondary: all-cause discontinuation, working alliance, adverse events, disorder-specific severity rating scales	Two-way video treatment was noninferior to face-to-face treatment in SF-36 MCS score (48.50 vs 46.68; p < 0.001); no significant differences in secondary outcomes
Milosevic et al. [12] (2022)	Canada	Non-randomized design	413	Adult outpatients with panic disorder/agoraphobia, social anxiety disorder, GAD, or OCD	Videoconference group CBT	Face-to-face group CBT	12 weekly sessions	Self-report measures of symptom outcomes and functional impairment	Face-to-face CBT showed only slight benefit over videoconference CBT; GAD group improved more in face-to-face; effect sizes were small; videoconference had slightly higher attendance rates in some instances; functional improvement and dropout rates were comparable
McCord et al. [13] (2022)	United States / Rural underserved communities	Prospective multi-site observational study	1,514	Patients receiving behavioral health treatment; nonrandomized convenience sample; telehealth and in-person groups; baseline demographics collected	Telehealth behavioral health treatment	In-person behavioral health treatment	One-month follow-up with monthly outcome measurement	PHQ-9 (depression), GAD-7 (anxiety)	Both telehealth and in-person groups showed similar improvements. PHQ-9 decreased by 2.8 (telehealth) vs 2.9 (in-person). GAD-7 decreased by 2.0 (telehealth) vs 2.4 (in-person). No significant difference between modalities. Higher baseline scores associated with greater improvement.
Bulkes et al. [14] (2022)	USA / Private nationwide behavioral health treatment system	Comparative cohort study (matched sample comparison)	2,384 total (1,192 telehealth; 1,192 in-person)	Patients receiving intensive psychological treatment for anxiety and depressive symptoms	Telehealth intensive psychological treatment during COVID-19	In-person intensive psychological treatment prior to COVID-19	Partial hospitalization noted; telehealth patients stayed longer	Depressive symptoms (QIDS-SR); Quality of life (Q-LES-Q)	No significant differences in depressive symptom reduction between groups; significant increases in quality of life across both groups; telehealth group had longer stay in partial hospitalization care.
Al-Alawi et al. [15] (2021)	Oman	Pragmatic Randomized Controlled Trial	46 (Intervention: 22, Control: 24)	Adults in Oman with symptoms of anxiety or depression during COVID-19 pandemic	Therapist-guided online therapy (weekly 1 session for 6 weeks; CBT + ACT; Arabic or English)	Self-help internet-based therapy (weekly email newsletter with CBT + ACT behavioral tips)	6 weeks / 1 session per week (intervention); weekly email (control)	PHQ-9 (depression), GAD-7 (anxiety)	Significant reduction in GAD-7 (β=−3.27; P=.01) and PHQ-9 scores (β=−4.311; P=.006) in intervention group compared to control; both groups showed improvement but therapist-guided online therapy was superior.
Comer et al. [16] (2017)	USA	RCT	22	Children aged 4–8 years with OCD	Videoteleconferencing (VTC)-delivered Family-Based CBT (FB-CBT)	Clinic-based FB-CBT	Not specified	Treatment retention, engagement, satisfaction, Clinical Global Impressions-Improvement Scale (CGI-I), symptom trajectories, family accommodation	High treatment retention, engagement, alliance, and satisfaction in both groups; at posttreatment, 72.7% VTC vs. 60% Clinic showed “excellent response”; at follow-up, 80% VTC vs. 66.7% Clinic showed “excellent response”; no significant differences between conditions
Lovell et al. [17] (2006)	United Kingdom / Two psychology outpatient departments	Randomised controlled non-inferiority trial	72	Patients with obsessive-compulsive disorder	Cognitive Behaviour Therapy (10 weekly sessions of exposure therapy and response prevention) delivered by telephone	Cognitive Behaviour Therapy delivered face-to-face	10 weekly sessions	Yale Brown Obsessive Compulsive Disorder Scale, Beck Depression Inventory, Client Satisfaction Questionnaire	Telephone-delivered CBT was equivalent to face-to-face CBT in clinical outcomes at 6 months; patient satisfaction was high in both groups.
Stubbings et al. [18] (2013)	Australia / University clinic	RCT	26	Primarily Caucasian clients; mean age 30 years (SD 11); diagnosed with mood or anxiety disorder (DSM-IV-TR)	CBT via videoconference	CBT in-person	12 sessions	Depression, Anxiety, Stress, Quality of Life, Working Alliance, Client Satisfaction	Significant reduction in depression, anxiety, stress, and improved quality of life in both groups (p<.001 no significant differences between videoconferencing and in-person cbt except stress reliable change favoring)
Turner et al. [19] (2014)	United Kingdom	Randomized Controlled Non-Inferiority Trial	72	Adolescents aged 11-18 years with primary OCD and their parents	TCBT with exposure and response prevention (E/RP), up to 14 sessions	Standard face-to-face CBT with E/RP, up to 14 sessions	Up to 14 sessions; assessment at mid-treatment, post-treatment, 3, 6, and 12-month follow-up	Children’s Yale–Brown Obsessive-Compulsive Scale (CY-BOCS), plus secondary measures	TCBT was not inferior to face-to-face CBT at post-treatment, 3-month, and 6-month follow-up; at 12 months no significant difference but CI exceeded non-inferiority threshold; improvements maintained through 12 months; high satisfaction in both groups

Efficacy of Telemedicine Interventions

Telemedicine interventions demonstrated comparable efficacy to in-person therapy across multiple studies. For instance, Rad et al. [10] found that internet-based CBT was as effective as face-to-face CBT in reducing social anxiety symptoms, with significant improvements maintained at a three-month follow-up. Similarly, Kishimoto et al. [11] reported non-inferior outcomes for two-way video treatment compared to in-person therapy, as measured by the SF-36 Mental Component Summary (MCS) score (48.50 vs. 46.68; p < 0.001). Milosevic et al. [12] observed small effect sizes favoring in-person CBT for GAD, but noted comparable functional improvements and attendance rates between videoconference and face-to-face groups.

In studies focusing on specific anxiety measures, McCord et al. [13] found no statistically significant difference in GAD-7 score reductions between telehealth and in-person groups (2.0 vs. 2.4 points, respectively). Likewise, Lovell et al. [17] and Turner et al. [19] established non-inferiority of telephone-delivered CBT for OCD, with no significant differences in clinical outcomes or long-term follow-up results. Stubbings et al. [18] further supported these findings, reporting significant reductions in anxiety symptoms for both videoconference and in-person CBT (p < .001), with no between-group differences (P = .41, d = 0.22).

Superiority in Specific Contexts

While most studies reported equivalent outcomes, therapist-guided telemedicine interventions showed superiority in certain contexts. Al-Alawi et al. [15] found that a six-week online CBT/ACT program led to greater reductions in GAD-7 (β = −3.27; p = 0.01) and PHQ-9 scores (β = −4.311; p = 0.006) compared to self-help internet-based therapy. Similarly, Comer et al. [16] noted higher rates of "excellent response" at follow-up for videoteleconferencing-delivered family-based CBT (80% vs. 66.7%), though the difference was not statistically significant.

Patient Engagement and Satisfaction

Telemedicine interventions were associated with high patient engagement and satisfaction. Bulkes et al. [14] reported longer stays in partial hospitalization care for telehealth patients, suggesting better adherence. Stubbings et al. [18] and Lovell et al. [17] highlighted comparable satisfaction levels between telemedicine and in-person groups, with both modalities achieving high client satisfaction scores.

Limitations and Heterogeneity

Despite the overall consistency in outcomes, some heterogeneity was noted. Milosevic et al. [12] observed slightly greater symptom improvement in face-to-face CBT for GAD, while Bulkes et al. [14] found no significant differences in depressive symptom reduction but noted telehealth patients had longer care durations. These variations may reflect differences in study design, participant characteristics, or intervention intensity (Table 2).

**Table 2:** Anxiety Outcomes across Included Studies

Author(s), Year	Intervention Group (Telemedicine)	Comparator Group (In-Person)	Outcome Measure	Mean (SD) or Change Score – Intervention	Mean (SD) or Change Score – Comparator	Effect Size / p-value
Rad et al. [10] (2024)	Internet-based CBT (10 weekly sessions)	Face-to-face CBT (10 weekly sessions)	Social phobia, fear of negative evaluation, social interaction anxiety, emotion regulation, physical symptoms, insomnia, social dysfunction, depression symptoms	ANCOVA showed significant reduction	ANCOVA showed significant reduction	Both interventions effective; significant improvement in both groups; effects stable at 3-month follow-up
Kishimoto et al. [11] (2024)	Two-way video treatment (≥50% video sessions)	Face-to-face treatment (100% in-person sessions)	SF-36 MCS score	48.50	46.68	p < 0.001 (non-inferior)
Milosevic et al. [12] (2022)	Videoconference group CBT for panic disorder/agoraphobia, social anxiety disorder, GAD, and OCD	Face-to-face group CBT for the same disorders	Symptom outcomes (validated self-report measures)	Slightly less improvement overall (exact means/SDs not reported); slightly higher attendance; comparable functional improvement and dropout	Slightly greater improvement overall; GAD group showed greater symptom improvement	Effect sizes small; only GAD difference significant
McCord et al. [13] (2022)	Telehealth behavioral health treatment	In-person behavioral health treatment	GAD-7 (Generalized Anxiety Disorder-7)	Reduction of 2.0 points (change score)	Reduction of 2.4 points (change score)	No statistically significant difference
Bulkes et al. [14] (2022)	Telehealth (N=1,192)	In-person (N=1,192)	Depressive symptoms (QIDS-SR), Quality of Life (Q-LES-Q)	Significant reduction in depressive symptoms; significant increase in QoL	Significant reduction in depressive symptoms; significant increase in QoL	No significant difference between groups
Al-Alawi et al. [15] (2021)	Therapist-guided online CBT/ACT therapy (1/week, 6 weeks)	Self-help internet-based CBT/ACT newsletters	GAD-7 (Anxiety) & PHQ-9 (Depression)	Greater reduction (β=−3.27 for GAD-7; β=−4.311 for PHQ-9)	Less reduction	GAD-7: p=0.01; PHQ-9: p=0.006
Comer et al. [16] (2017)	VTC-delivered Family-Based CBT (FB-CBT)	Clinic-based FB-CBT	“Excellent response” (1 or 2 on Clinical Global Impressions-Improvement Scale) at posttreatment and 6-month follow-up	Post: 72.7%, Follow-up: 80%	Post: 60%, Follow-up: 66.7%	No significant difference reported
Lovell et al. [17] (2006)	Telephone-delivered CBT (10 sessions)	Face-to-face CBT (10 sessions)	Yale-Brown OCD Scale	Difference: −0.55 (95% CI −4.26 to 3.15)	Reference group	No significant difference; non-inferiority established
Stubbings et al. [18] (2013)	CBT via videoconference	CBT in-person	Anxiety symptoms	Significant reduction (no specific mean (SD) reported)	Significant reduction (no specific mean (SD) reported)	No significant difference between groups (P = .41, d = 0.22)
Turner et al. [19] (2014)	Telephone CBT (TCBT)	Face-to-face CBT	CY-BOCS	TCBT not inferior at post, 3m, 6m	No significant difference	Non-inferior at post, 3m, 6m; 12m no significant difference

Results of Risk of Bias Assessment

The Cochrane RoB 2 tool was used to assess the risk of bias in the seven RCTs included in this review. Six studies [10, 11, 15, 17-19] were rated as low risk across all domains, indicating robust methodology with proper randomization, minimal deviations from interventions, complete outcome data, and no selective reporting. However, Comer et al. [16] was rated as high risk due to a small sample size (n=22) and potential attrition bias, despite low concerns in other domains (Table 3).

**Table 3:** Cochrane RoB 2 Tool Risk of Bias Assessment

Study	Randomization Bias	Deviations from Intended Interventions	Missing Outcome Data	Outcome Measurement Bias	Selective Reporting	Overall Risk
Rad et al. [10] (2024)	Low	Low	Low	Low	Low	Low
Kishimoto et al. [11] (2024)	Low	Low	Low	Low	Low	Low
Al-Alawi et al. [15] (2021)	Low	Low	Low	Low	Low	Low
Comer et al. [16] (2017)	Some concerns	Low	High	Low	Low	High
Lovell et al. [17] (2006)	Low	Low	Low	Low	Low	Low
Stubbings et al. [18] (2013)	Low	Low	Low	Low	Low	Low
Turner et al. [19] (2014)	Low	Low	Low	Low	Low	Low

For the three non-randomized studies, the NOS was applied. Milosevic et al. [12] and Bulkes et al. [14] were rated as low risk, with high scores in selection, comparability, and outcome assessment. McCord et al. [13] received a moderate risk rating due to its observational design and potential confounding factors, though it still maintained reasonable methodological quality (Table 4).

**Table 4:** Newcastle Ottawa Scale (NOS) Tool Risk of Bias Assessment

Study	Selection (Max 4)	Comparability (Max 2)	Outcome (Max 3)	Total (Max 9)	Risk of Bias
Milosevic et al. [12] (2022)	3	1	3	7	Low
McCord et al. [13] (2022)	3	1	2	6	Moderate
Bulkes et al. [14] (2022)	4	2	3	9	Low

Discussion

The findings of this systematic review suggest that telemedicine-delivered psychological interventions can be as effective as in-person therapy for treating anxiety disorders, although this conclusion must be interpreted with caution due to considerable heterogeneity across studies. The 10 included studies employed a range of modalities - such as internet-based CBT, videoconferencing, telephone-delivered therapy, and two-way video sessions - with variable levels of therapist involvement, intensity, and session length. Although Rad et al. [10] and Kishimoto et al. [11] reported comparable improvements between telemedicine and face-to-face groups, the interventions they assessed differ substantially in content and delivery. Thus, while non-inferiority was demonstrated in specific studies, these findings cannot be universally applied across all telehealth formats without further stratification. This aligns with existing meta-analytic evidence [20, 21], which supports digital CBT's core therapeutic mechanisms but also underscores the importance of delivery context.

Therapist-guided telemedicine emerged as particularly effective, especially when compared with self-help interventions. For example, Al-Alawi et al. [15] showed significantly better outcomes for guided online CBT/ACT versus self-guided newsletters, reaffirming the need for clinician support in maximizing digital treatment efficacy. This is consistent with prior research [22] emphasizing the role of professional guidance in improving adherence and reducing dropout rates. Similarly, Comer et al. [16] found stronger treatment responses in a videoteleconferencing-based family CBT for pediatric OCD, although its small sample size and high risk of bias limit the strength of this finding. These examples highlight how intervention intensity and therapist involvement are key moderators of treatment success-factors that future reviews should structure into analytic subgroups [23].

Patient engagement and satisfaction were generally high across modalities, suggesting telemedicine is an acceptable alternative for many. For instance, Bulkes et al. [14] reported longer retention among telehealth patients in partial hospitalization care, possibly due to greater convenience or flexibility - an important consideration given high dropout rates in standard care [24]. Lovell et al. [17] and Stubbings et al. [18] also demonstrated comparable satisfaction between remote and in-person therapy, echoing patient preferences observed in recent surveys [25]. However, such comparisons risk oversimplification unless demographic and contextual factors - such as digital access, literacy, and regional disparities - are explicitly examined, as done by McCord et al. [13] in rural settings.

Importantly, the variability in outcomes across disorders and modalities was notable. For example, Milosevic et al. [12] observed slightly larger symptom reductions in face-to-face CBT for GAD, although the differences were modest. This finding is mirrored in meta-analyses [26] reporting marginal advantages for in-person care, potentially due to non-verbal cues or stronger therapeutic alliance. Conversely, Bulkes et al. [14] found no significant differences in depressive symptom reduction but longer treatment durations for telehealth, hinting at potential "digital dose" effects. Such variability underlines the need for subgroup analyses by treatment intensity, delivery mode, and patient characteristics to make more actionable conclusions.

Although risk of bias assessments were conducted, their integration into the interpretation of results was limited. Most RCTs, including those by Rad et al. [10] and Kishimoto et al. [11], were rated low risk, lending confidence to their findings. However, the conclusions also drew upon studies with notable methodological limitations. Comer et al. [16], for instance, had a high risk of bias due to a small sample and potential attrition. Likewise, non-randomized studies such as McCord et al. [13] and Milosevic et al. [12] had moderate risks due to confounding, yet were discussed with equal weight. A more structured weighting based on study quality would improve the robustness of synthesis [27].

Equity and access considerations, though briefly acknowledged, were not deeply analyzed in this review. Digital disparities - such as internet availability, device access, and digital literacy - remain critical in determining who benefits from telehealth and who is left behind. For example, while McCord et al. [13] provided insights from rural populations, no included study disaggregated results by socioeconomic status, race/ethnicity, or age group. This limits the generalizability and practical relevance of the findings, especially in designing inclusive digital mental health strategies [28].

The COVID-19 pandemic undeniably catalyzed telehealth adoption, and several studies - including Al-Alawi et al. [15] and McCord et al. [13] - showed its utility in crisis conditions. Others, like Kishimoto et al. [11] and Turner et al. [19], support telemedicine’s sustained effectiveness for chronic mental health needs. This evolution reflects real-world data [29] showing that telehealth can reduce access barriers. Yet, its long-term viability requires addressing digital inequities and ensuring culturally competent delivery across diverse populations.

Limitations

This review was limited by heterogeneity in intervention formats, comparator definitions, and outcome measures (e.g., GAD-7, SF-36 MCS), which complicates cross-study comparison despite some consistent trends. Most included studies featured short follow-up periods, limiting insights into long-term effectiveness. Furthermore, the review did not conduct subgroup analyses by intervention type, delivery intensity, or patient demographics, which would have improved analytical depth. Cultural and socioeconomic diversity was also limited, with most studies from high-income settings. Lastly, while a risk of bias assessment was performed, its impact was not formally weighted in the interpretation, and publication bias remains a concern.

## Conclusions

Telemedicine appears to be a promising alternative to in-person therapy for anxiety disorders, particularly in improving accessibility and patient satisfaction, especially when delivered with therapist guidance. However, given the considerable variability in intervention types, delivery formats, and study quality, conclusions regarding its equivalence to in-person care should be drawn with caution. The current evidence supports the potential integration of telehealth into mental health services, but its implementation should be tailored to individual needs and contextual factors. Future research should focus on long-term outcomes, subgroup analyses, and equity-focused delivery models to better inform clinical practice and policy decisions.

## Appendices

**Table 5:** Search Strategy for Databases

Database	Search Strategy	Date Searched
PubMed	(“Telemedicine”[MeSH] OR telemedicine OR telehealth OR telepsychology OR teletherapy OR “online therapy” OR “internet-based therapy” OR videoconferencing) AND (“Anxiety Disorders”[MeSH] OR anxiety OR “generalised anxiety disorder” OR GAD OR panic OR “social anxiety disorder” OR phobia) AND (“psychological intervention” OR psychotherapy OR CBT OR “cognitive behavioural therapy” OR therapy)	July 2025
Scopus	TITLE-ABS-KEY (telemedicine OR telehealth OR telepsychology OR teletherapy OR “online therapy” OR “internet-based therapy” OR videoconferencing) AND TITLE-ABS-KEY (anxiety OR “anxiety disorder” OR GAD OR panic OR “social anxiety disorder” OR phobia) AND TITLE-ABS-KEY (psychotherapy OR “psychological intervention” OR CBT OR “cognitive behavioural therapy” OR therapy)	July 2025
Web of Science	TS=(telemedicine OR telehealth OR telepsychology OR teletherapy OR “online therapy” OR “internet-based therapy” OR videoconferencing) AND TS=(anxiety OR “anxiety disorder” OR GAD OR panic OR “social anxiety disorder” OR phobia) AND TS=(psychotherapy OR “psychological intervention” OR CBT OR “cognitive behavioural therapy” OR therapy)	July 2025
ClinicalTrials.gov	Condition or disease: Anxiety OR “Anxiety Disorder”	Other terms: telemedicine OR telehealth OR telepsychology OR teletherapy OR online therapy OR videoconferencing

## References

[1] Javaid SF, Hashim IJ, Hashim MJ, Stip E, Samad MA, Ahbabi AA (2023). Epidemiology of anxiety disorders: global burden and sociodemographic associations. Middle East Curr Psychiatry.

[2] Yang X, Fang Y, Chen H (2021). Global, regional and national burden of anxiety disorders from 1990 to 2019: results from the Global Burden of Disease Study 2019. Epidemiol Psychiatr Sci.

[3] Xiong P, Liu M, Liu B, Hall BJ (2022). Trends in the incidence and DALYs of anxiety disorders at the global, regional, and national levels: estimates from the Global Burden of Disease Study 2019. J Affect Disord.

[4] Shaker AA, Austin SF, Storebø OJ (2023). Psychiatric treatment conducted via telemedicine versus in-person modality in posttraumatic stress disorder, mood disorders, and anxiety disorders: systematic review and meta-analysis. JMIR Ment Health.

[5] Omboni S, Padwal RS, Alessa T (2022). The worldwide impact of telemedicine during COVID-19: current evidence and recommendations for the future. Conn Health Telemed.

[6] Greenwood H, Krzyzaniak N, Peiris R (2022). Telehealth versus face-to-face psychotherapy for less common mental health conditions: systematic review and meta-analysis of randomized controlled trials. JMIR Ment Health.

[7] Page MJ, McKenzie JE, Bossuyt PM (2021). The PRISMA 2020 statement: an updated guideline for reporting systematic reviews. BMJ.

[8] Sterne JA, Savović J, Page MJ (2019). RoB 2: a revised tool for assessing risk of bias in randomised trials. BMJ.

[9] Wells GA, Shea B, O’Connell D, Peterson J, Welch V, Losos M, Tugwell P (2000). The Newcastle-Ottawa Scale (NOS) for Assessing the Quality of Nonrandomised Studies in Meta-Analyses. Ottawa Hospital Res Institute.

[10] Rad HS, Goodarzi H, Bahrami L, Abolghasemi A (2024). Internet-based versus face-to-face cognitive-behavioral therapy for social anxiety disorder: a randomized control trial. Behav Ther.

[11] Kishimoto T, Kinoshita S, Kitazawa M (2024). Live two-way video versus face-to-face treatment for depression, anxiety, and obsessive-compulsive disorder: a 24-week randomized controlled trial. Psychiatry Clin Neurosci.

[12] Milosevic I, Cameron DH, Milanovic M, McCabe RE, Rowa K (2022). Face-to-face versus video teleconference group cognitive behavioural therapy for anxiety and related disorders: a preliminary comparison. Can J Psychiatry.

[13] McCord C, Ullrich F, Merchant KA (2022). Comparison of in-person vs. telebehavioral health outcomes from rural populations across America. BMC Psychiatry.

[14] Bulkes NZ, Davis K, Kay B, Riemann BC (2022). Comparing efficacy of telehealth to in-person mental health care in intensive-treatment-seeking adults. J Psychiatr Res.

[15] Al-Alawi M, McCall RK, Sultan A (2021). Efficacy of a six-week-long therapist-guided online therapy versus self-help internet-based therapy for COVID-19-induced anxiety and depression: open-label, pragmatic, randomized controlled trial. JMIR Ment Health.

[16] Comer JS, Furr JM, Kerns CE (2017). Internet-delivered, family-based treatment for early-onset OCD: a pilot randomized trial. J Consult Clin Psychol.

[17] Lovell K, Cox D, Haddock G (2006). Telephone administered cognitive behaviour therapy for treatment of obsessive compulsive disorder: randomised controlled non-inferiority trial. BMJ.

[18] Stubbings DR, Rees CS, Roberts LD, Kane RT (2013). Comparing in-person to videoconference-based cognitive behavioral therapy for mood and anxiety disorders: randomized controlled trial. J Med Internet Res.

[19] Turner CM, Mataix-Cols D, Lovell K, Krebs G, Lang K, Byford S, Heyman I (2014). Telephone cognitive-behavioral therapy for adolescents with obsessive-compulsive disorder: a randomized controlled non-inferiority trial. J Am Acad Child Adolesc Psychiatry.

[20] Andrews G, Basu A, Cuijpers P, Craske MG, McEvoy P, English CL, Newby JM (2018). Computer therapy for the anxiety and depression disorders is effective, acceptable and practical health care: an updated meta-analysis. J Anxiety Disord.

[21] Carlbring P, Andersson G, Cuijpers P, Riper H, Hedman-Lagerlöf E (2018). Internet-based vs. face-to-face cognitive behavior therapy for psychiatric and somatic disorders: an updated systematic review and meta-analysis. Cogn Behav Ther.

[22] Andersson G, Titov N, Dear BF, Rozental A, Carlbring P (2019). Internet-delivered psychological treatments: from innovation to implementation. World Psychiatry.

[23] Titov N, Dear BF, Staples LG (2017). The first 30 months of the MindSpot Clinic: evaluation of a national e-mental health service against project objectives. Aust N Z J Psychiatry.

[24] Fernandez E, Woldgabreal Y, Day A, Pham T, Gleich B, Aboujaoude E (2021). Live psychotherapy by video versus in-person: a meta-analysis of efficacy and its relationship to types and targets of treatment. Clin Psychol Psychother.

[25] Wind TR, Rijkeboer M, Andersson G, Riper H (2020). The COVID-19 pandemic: the 'black swan' for mental health care and a turning point for e-health. Internet Interv.

[26] Varker T, Brand RM, Ward J, Terhaag S, Phelps A (2019). Efficacy of synchronous telepsychology interventions for people with anxiety, depression, posttraumatic stress disorder, and adjustment disorder: a rapid evidence assessment. Psychol Serv.

[27] Himelein-Wachowiak M, Giorgi S, Devoto A (2021). Bots and misinformation spread on social media: implications for COVID-19. J Med Internet Res.

[28] Zhou X, Snoswell CL, Harding LE, Bambling M, Edirippulige S, Bai X, Smith AC (2020). The role of telehealth in reducing the mental health burden from COVID-19. Telemed J E Health.

[29] Pierce RP, Stevermer JJ (2023). Disparities in the use of telehealth at the onset of the COVID-19 public health emergency. J Telemed Telecare.

